# Improvement of the Anticancer Efficacy of PD‐1/PD‐L1 Blockade: Advances in Molecular Mechanisms and Therapeutic Strategies

**DOI:** 10.1002/mco2.70274

**Published:** 2025-07-15

**Authors:** Peng Gao, Xiao Li, Zhenyu Duan, Yang Wang, Yinggang Li, Jing Wang, Kui Luo, Jie Chen

**Affiliations:** ^1^ Department of General Surgery Breast Disease Center Department of Radiology Huaxi MR Research Center (HMRRC) Institution of Radiology and Medical Imaging Sichuan Engineering Research Center for Intelligent Diagnosis and Treatment of Breast Diseases Frontiers Science Center for Disease‐Related Molecular Network State Key Laboratory of Biotherapy West China Hospital Sichuan University Chengdu China; ^2^ Department of Anus and Intestine Surgery First Affiliated Hospital of China Medical University Shenyang China; ^3^ Functional and Molecular Imaging Key Laboratory of Sichuan Province, and Research Unit of Psychoradiology, Chinese Academy of Medical Sciences Chengdu China; ^4^ Department of Medical Oncology National Cancer Center/National Clinical Research Center for Cancer/Cancer Hospital Chinese Academy of Medical Sciences and Peking Union Medical College Beijing China; ^5^ Department of Breast Surgical Oncology, National Cancer Center/National Clinical Research Center for Cancer/Cancer Hospital Chinese Academy of Medical Sciences and Peking Union Medical College Beijing China

**Keywords:** PD‐1/PD‐L1 blockade resistance, cancer treatment, type I interferons, cGAS‐STING pathway

## Abstract

The clinical success of PD‐1/PD‐L1 blockade has revolutionized cancer immunotherapy. However, the issues of immune resistance have become increasingly prominent, representing a critical limitation in modern oncology. This phenomenon has prompted efforts to elucidate the mechanisms underlying both types of resistance and to find breakthrough therapeutic strategies. This article provides a comprehensive overview of PD‐1/PD‐L1 blockade resistance mechanisms from both primary and acquired resistance perspectives, including tumor intrinsic factors, immune microenvironment components, and systemic factors. Building on this foundation, emerging research demonstrates that type I interferons (IFNs), particularly IFN‐α and IFN‐β, play crucial immunomodulatory roles in overcoming resistance to PD‐1/PD‐L1 blockade. We delineate six molecular mechanisms through which IFN‐α/β enhance PD‐1/PD‐L1 blockade efficacy, and innovative strategies are proposed to therapeutically boost IFN‐α/β production, including gene editing techniques, targeting the cGAS‐STING or TLR pathway and so on. Furthermore, insights into current challenges and future directions of the application of IFN‐α/β to improve PD‐1/PD‐L1 blockade are discussed. This review holds significant academic value by not only synthesizing current knowledge on PD‐1/PD‐L1 resistance mechanisms but also pioneering a framework for leveraging type I IFNs to overcome these barriers.

## Graphical abstract

Mechanisms of alleviating resistance to PD‐1/PD‐L1 blockade by IFN‐α and IFN‐β. In these mechanisms, the “yin‐yang balance” is disrupted, thus preventing T cells from attacking cancer cells. Restoring this balance may improve the efficacy of anti‐PD‐1/PD‐L1 therapy and reinvigorate T cells and other immune cells to target and destroy cancer cells.

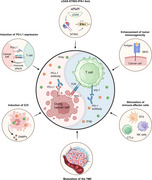

## 1 Introduction

The domain of oncological immunotherapy has entered a transformative era, distinguished by the advancement of immune checkpoint inhibitors (ICIs) [1, 2]. Among these immune checkpoints, the PD‐1/PD‐L1 pathway has shown great promise as a therapeutic target. In 1992, Honjo et al. [3] discovered programmed cell death protein 1 (PD‐1). It was demonstrated that PD‐1 knockout in the mice led to autoimmune diseases and abnormal activation of immune cells, underscoring its critical function in modulating immune responses. In 1999, Chen et al. [4] at Yale University identified B7‐H1, later termed PD‐L1, as a ligand for PD‐1, revealing the mechanism by which tumors hijack the PD‐1/PD‐L1 axis to escape immune detection. The approval of ICIs for cancer treatment marked a significant breakthrough in cancer therapy. In July 2014, the first PD‐1 inhibitor, Nivolumab, was granted marketing approval in Japan for the treatment of melanoma [5]. Shortly thereafter, the Food and Drug Administration (FDA) of America authorized Pembrolizumab, another PD‐1 inhibitor in September 2014 [6].

In the last 10 years, the clinical application of these ICIs, particularly those targeting the PD‐1/PD‐L1 axis, has fundamentally transformed the approach to treating advanced cancers, particularly solid tumors [7, 8, 9]. This progress has spurred the establishment of immuno‐oncology departments in academic institutions, biopharmaceutical companies, and hospitals [10, 11]. Despite these advances, primary resistance to anti‐PD‐1 therapy has become prevalent, affecting as many as 60% of individuals diagnosed with specific cancer subtypes [5]. Moreover, increasing evidence suggests initial positive responses in some patients may be undermined by the acquired resistance, leading to disease progression or relapse [12, 13]. This phenomenon has prompted efforts to elucidate the mechanisms underlying both types of resistance. Resistance to anti‐PD‐1/PD‐L1 therapy is broadly categorized into primary and acquired forms [10]. Primary resistance is frequently attributed to tumor‐intrinsic factors, including insufficient expression of tumor‐associated antigens [14], modifications within the tumor microenvironment (TME), dysfunctions in local immune signaling pathways, as well as the patient's systemic conditions [15, 16]. Acquired resistance may be associated with broader biological influences, including intrinsic tumor factors, influences of other immune checkpoints, alterations in T cells, and so on, and they have been demonstrated to significantly impact therapeutic responses [17, 18, 19]. In response to these complexities, researchers have investigated approaches to improve the efficacy of anti‐PD‐1/PD‐L1 therapy. A key strategy involves designing combination therapies that pair immunomodulatory agents with other treatments for manipulating immune regulatory pathways, thereby aiming to improve immune responses and mitigate resistance development during cancer immunotherapy [20, 21, 22]. For example, anti‐PD‐1/PD‐L1 therapy could be combined with other ICIs, targeted therapy, chemotherapy, and/or radiotherapy [23, 24, 25, 26]. These combinations aim to synergistically boost immune responses and overcome resistance by reshaping the TME and strengthening tumor antigenicity. Another strategy involves modulating PD‐L1 expression. Manipulating key signaling cascades, such as PI3K‐AKT and EGFR pathways, and employing epigenetic modifications to regulate PD‐L1 levels could sensitize tumors to checkpoint blockade [27, 28]. Novel approaches to using oncolytic viral therapies and adoptive cell‐based treatments have demonstrated the ability to reshape the tumor microenvironment and promote increased T‐cell infiltration [29, 30, 31]. Targeting immunosuppressive elements and altering the extracellular matrix in the TME can also contribute to revitalizing PD‐1/PD‐L1 inhibitor activity against tumors [32, 33, 34].

Through a comprehensive literature review, type I IFNs have been identified as critical regulators influencing the efficacy of anti‐PD‐1/PD‐L1 treatments [35, 36]. These cytokines, encompassing various IFN subtypes (IFNα, IFNβ, IFNε, IFNω, and IFNκ), play a pivotal role in orchestrating immune system activation and modulation, particularly in the processes of tumor development and malignant progression [37, 38]. Research suggests that type I IFNs, particularly IFN α/β, are crucial for enhancing the effectiveness of anti‐PD‐1/PD‐L1 therapies, and even for overcoming resistance to these ICIs [39, 40]. Revealing the interaction between type I IFNs signaling and the PD‐1/PD‐L1 axis is vital for developing innovative approaches to address resistance in cancer immunotherapy.

However, very few review articles have been devoted to addressing the contributions of IFN‐α and IFN‐β in overcoming resistance to anti‐PD‐1/PD‐L1 therapy. In this review, we first summarized the unveiled mechanisms of PD‐1/PD‐L1 blockade resistance in terms of both primary and acquired types and elaborated primary sources for IFN‐α/β production. We then discussed cutting‐edge strategies leveraging IFN‐α and IFN‐β to address both primary and acquired resistance to anti‐PD‐1/PD‐L1 therapy and summarized clinical trials of IFN‐α/β integrated with anti‐PD‐1/PD‐L1 treatments. Finally, we conducted an in‐depth analysis of the current challenges of these strategies and proposed future directions for research and clinical development. This review article will provide impactful insights into exploring the potential of IFN‐α/β for preclinical and clinical development of therapeutic methods for improving PD‐1/PD‐L1 blockade resistance.

## 2 Mechanisms of PD‐1/PD‐L1 Blockade Resistance

**FIGURE 1:**
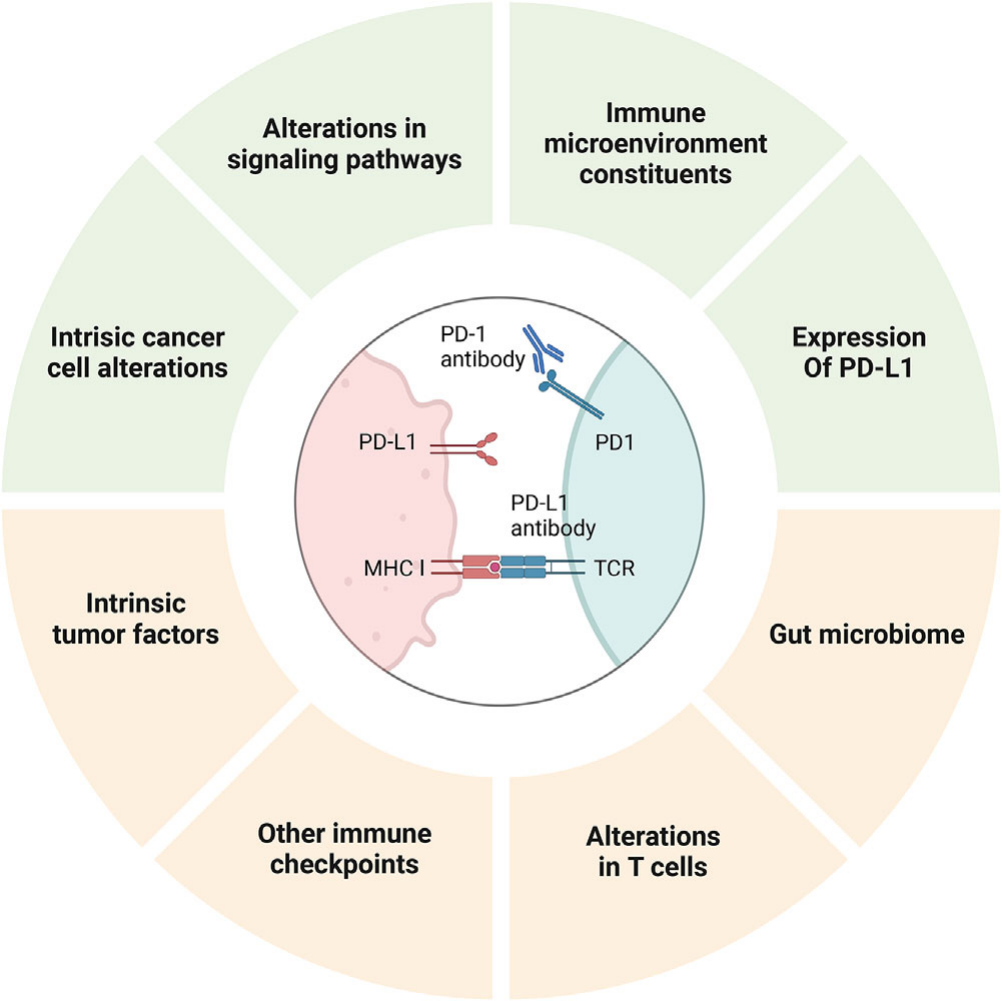
Diagrammatic representation of the mechanisms of PD‐1/PD‐L1 blockade resistance. The upper section illustrates the mechanisms of primary resistance, while the lower section depicts the mechanisms of acquired resistance. Created with BioRender.com.

Cancer immunotherapy, particularly with PD‐1/PD‐L1 antibodies, has demonstrated remarkable clinical success in treating solid tumors recently, marking a groundbreaking advancement in oncology, offering renewed hope and a potential turning point for patients battling cancer [41, 42, 43]. However, a portion of cancer patients exhibit treatment resistance at different stages during therapy, ultimately leading to tumor recurrence and progression. Therefore, the development of resistance represents a ‘roadblock’ to the continued benefit of immunotherapy [44, 45, 46]. Gaining a more comprehensive understanding of the mechanisms governing resistance to PD‐1/PD‐L1 blockade and identifying approaches to counteract immunotherapy resistance are urgent priorities for the precision immunotherapy of cancer [47, 48]. Figure 1 comprehensively illustrates the mechanisms underlying both primary and acquired resistance to PD‐1/PD‐L1 blockade therapy.

### 2.1 Primary Resistance

#### 2.1.1 Intrinsic Cancer Cell Alterations

The ability of effector T cells to identify tumor antigens is a critical factor in cancer therapy. Alterations in tumor antigen profiles or their levels can impact both T cell recognition and their cytotoxic function [49, 50]. Tumor mutational burden (TMB) is positively correlated with mutation‐associated neoantigens. Research has demonstrated that TMB is significantly elevated in renal cell carcinoma, non‐small‐cell lung cancer (NSCLC), and melanoma, compared with pancreatic cancer and prostate cancer [51, 52, 53]. The cancer types with a high TMB exhibit greater sensitivity to anti‐PD‐1/PD‐L1 therapy. Moreover, deficiencies in DNA mismatch repair (dMMR) and high microsatellite instability (MSI‐H) are frequently detected in diverse cancers, driving an increase in the generation of tumor‐associated neoantigens, which may be beneficial to ICI therapy. By contrast, a low tumor mutational burden, an enhancement in dMMR, or low microsatellite instability can lead to decreased generation of tumor‐associated antigens, potentially contributing to the development of resistance [54].

#### 2.1.2 Alterations in Signaling Pathways

Regulation of tumor‐endogenous antigen signaling pathways can influence tumor antigen expression, hindering T‐cell recognition [55, 56, 57]. For instance, activation of the MAPK pathway promotes the secretion of IL‐8 and VEGF, which inhibit T cell recruitment and function, thereby limiting the infiltration of effector T cells (Teffs) into the tumor microenvironment [58]. Research has shown that the tumor suppressor gene PTEN is often inactivated in multiple cancer types [59]. Loss of PTEN function amplifies PI3K signaling, leading to decreased expression of granzyme B, reduced interferon‐γ (IFN‐γ) production, and diminished infiltration of CD8+ T cells within the tumor. In melanoma, PTEN gene loss is associated with reduced T cell infiltration into tumors, impaired T cell proliferation after tumor removal, and poorer responses to PD‐1 blockade therapies [60].

The IFN‐γ signaling pathway plays a dual role in cancer immunity, contributing to both primary and acquired resistance mechanisms [15, 61]. IFN‐γ released by tumor‐specific T cells can identify tumor cells and their associated antigens, triggering the upregulation of proteins such as effector molecules and major histocompatibility complex (MHC) proteins that inhibit tumor growth or promote apoptosis. Therefore, tumors exhibiting defective IFN‐γ signaling pathways are more resistant to T cell‐mediated elimination, fostering resistance to ICIs [62, 63]. On the other hand, chronic IFN‐γ exposure can drive tumor cell reprogramming, facilitating immune escape mechanisms [64].

#### 2.1.3 Immune Microenvironmental Constituents

Numerous immune cells frequently infiltrate and populate the tumor microenvironment, collectively establishing a protective barrier against the malignancy [65, 66, 67]. However, when this barrier is compromised, immunosuppressive cells are predominantly present within the TME, playing a critical role in promoting tumor growth and progression. Immunosuppressive cells primarily consist of tumor‐associated macrophages (TAMs), regulatory T cells (Tregs), and myeloid‐derived suppressor cells (MDSCs). These cells suppress the activation, proliferation, and cytotoxic activity of CD8+ T cells through mechanisms such as expressing co‐inhibitory receptors and releasing immunosuppressive cytokines, ultimately enabling tumor cells to evade immune detection [68, 69, 70].

Moreover, inhibitory cytokines present within the TME significantly contribute to immunotherapy resistance. These immunosuppressive factors, often produced by tumor cells or macrophages, suppress the body's anticancer immune responses [71, 72, 73]. Tumor cells secrete chemokines like CCL5, CCL7, and CXCL8, which recruit Treg cells and MDSCs into TME. These immune suppressive cells produce immunosuppressive cytokines like TGF‐β and IL‐6, which disrupt CD8+ T cell activity and induce resistance to immunotherapy [74, 75].

#### 2.1.4 Expression of PD‐L1

Tumors can express PD‐L1 on their surface, which effectively inhibits the antitumor activity of T cells. The PDJ (PD‐L1‐PD‐L2‐JAK2) amplicon is a gene located on chromosome 9, containing two ligands of PD‐1 (PD‐L1 and PD‐L2) as well as JAK2, an INF‐γ receptor signaling molecule. As a result, the expression of PDJ can contribute to an elevated PD‐L1 level [76]. Studies have demonstrated that alterations in the constitutive expression of PD‐L1 in cancer cells can be driven by various genetic abnormalities, including PTEN loss, PI3K or AKT mutations, EGFR mutations, MYC overexpression, CDK5 dysregulation, and PD‐L1 gene mutations or deletions [77].

### 2.2 Acquired Resistance

#### 2.2.1 Intrinsic Tumor Factors

Recent studies have unveiled a few mechanisms of intrinsic acquired resistance in tumors, which may be involved in mutations affecting the IFN signaling pathway, modifications of target antigens, and structural alterations in human leukocyte antigen 1 (HLA‐1) molecules [78, 79]. These changes are often associated with dysfunctions in antigen presentation or a reduction in antigen expression, leading to failures in recognizing tumor cells by T cells. During ICI treatment, pre‐existing mutations may disappear, while more complex de novo mutations arise, along with a shift in the T cell repertoire, therefore, cancer cells could bypass immune detection independently of the PD‐1 pathway, contributing to ICI resistance [79]. For example, mutations in β‐2 microglobulin (β‐2M) on cancer cell surfaces can result in the loss of HLA‐1 expression, preventing the effective presentation of tumor antigens on the cell surface, thereby hindering CD8+ T cell recognition [80].

#### 2.2.2 Other Immune Checkpoints

Beyond the commonly recognized PD‐1, PD‐L1, and CTLA‐4 immune checkpoints, recent studies have identified other checkpoints, such as LAG‐3, TIGIT, VISTA, and TIM‐3, and unknown checkpoints are currently under investigation [80]. Inhibiting specific immune checkpoints with ICIs can lead to the compensatory activation of alternative immune checkpoints, thereby contributing to resistance [81]. For instance, combining CTLA‐4 inhibitors with PD‐1/PD‐L1 antibodies can activate the INF‐γ pathway and other pathways, potentially inducing the upregulation of additional immune checkpoints on tumor‐infiltrating lymphocytes, ultimately resulting in treatment failure. Reports indicate that in two lung adenocarcinoma patients experiencing cancer progression following PD‐1 inhibitor therapy, pleural effusions contained a high proportion of TIM‐3^+^ T cells [82].

#### 2.2.3 Alterations in T Cells

Another potential mechanism of tumor recurrence is the functional loss of T cells, which impairs the efficient expression of tumor antigens. Furthermore, prolonged anticancer activity can cause T‐cell exhaustion, ultimately reducing the T‐cell population within the TME [83, 84]. These tumors, often described as “cold tumors,” are featured with a “deserted” tumor microenvironment. In an extreme case of T cell exhaustion, acquired immune resistance may develop.

#### 2.2.4 Gut Microbiome

The gut microbiome is commonly coined as the “second genome” of humans [85]. It plays a key role in the initiation and progression of various cancers, including gastric, colorectal, and liver cancer, through mechanisms like triggering chronic inflammation, secreting toxins, and altering hormone levels. Previous studies have found that the microbiome can influence the effectiveness of immunotherapy [86, 87, 88]. The patients with gut microbiomes enriched in *Bifidobacterium* and *Enterococcus* most likely benefit from immunotherapy [89]. The transplantation of *Bacteroides fragilis* into patients has been shown to enhance tumor responsiveness to CTLA‐4 inhibitors [90]. In melanoma, a reduction in the gut microbiome diversity is closely linked to poor treatment outcomes after PD‐1 blockade [91]. Additionally, antibiotic use can disrupt the balance of the gut microbiome, potentially contributing to the development of resistance to immunotherapy [91].

Cancer exhibits highly complex, dynamic, and heterogeneous biological characteristics. Tumor intrinsic factors, the TME, and patient‐specific systemic elements collectively drive resistance to anti‐PD‐1/PD‐L1 therapies [92, 93, 94]. In the field of ICI therapy, revealing the intricate mechanisms behind PD‐1/PD‐L1 blockade resistance and developing approaches to improving the precise targeting of this treatment are two hot topics. A thorough review of the latest literature highlights the pivotal role of type I IFNs, particularly IFN‐α and IFN‐β, in enhancing the efficacy of PD‐1/PD‐L1 blockade. IFN‐α and IFN‐β are essential regulators in overcoming both primary and secondary resistance to anti‐PD‐1/PD‐L1 therapy. Therefore, we will delve into detailed mechanisms through which IFN‐α and IFN‐β can counteract PD‐1/PD‐L1 blockade resistance in the following sections. Additionally, we will explore how these cytokines can be leveraged to develop cutting‐edge strategies for improving immune resistance in cancer therapy.

## 3 The Role of Type I IFNs IN Cancer Therapy

Type I IFNs, discovered over half a century ago, are a family of widely expressed cytokines. They are critical for antiviral defense and immune modulation when the body is challenged by viral infections [95, 96]. The human type I IFNs family comprises 18 members, including 13 IFN‐α subtypes, IFN‐β, IFN‐ε, IFN‐ĸ, and IFN‐ω. Among them, IFN‐α and IFN‐β are pivotal, with their genes located on the same chromosome [97, 98]. While IFN‐α subtypes are highly diverse and encoded by separate genes, IFN‐β is derived from a single gene, highlighting its unique and nonredundant role [99, 100]. Many cells have the ability to produce type I IFNs, but a large quantity of them are typically produced by plasmacytoid dendritic cells (pDCs) [101].

Emerging research indicates that type I IFNs play a critical regulatory function in immune system modulation, malignant cell identification, and T lymphocyte activation, including (1) cancer cell‐intrinsic effects: in malignant cells, the type I IFN signaling pathway mediates both cytostatic and cytotoxic by upregulating cell cycle inhibitors including cyclin‐dependent kinase inhibitor 1A (CDKN1A) [102], CDKN1B [103], and CDKN2D [104], as well as downregulating proteins essential for cell cycle progression including cyclin D3 (CCND3) and cell division cycle 25A (CDC25A) [105]; (2) regulation of the cell cycle: abnormal cell division in malignant tumors is usually accompanied with mutations in cell cycle regulatory genes, leading to overexpression of growth‐initiating proteins. Studies have revealed that type I interferons (IFNs) can interfere with the progression of malignant cells through the cell cycle, particularly by impeding the G0‐G1 phase transition [106, 107]; (3) induction of apoptosis/pyroptosis: apoptosis serves as a regulated, noninflammatory cellular demise mechanism for eliminating damaged or aberrant cells in the tumor tissue, while pyroptosis triggers inflammatory responses and immune activation, counteracting tumor growth and dissemination. Type I IFNs a possess the capacity to induce apoptosis in malignant cells via through exogenous pathways mediated by apoptosis‐inducing ligands, or pyroptosis through the depletion of ubiquitin specific proteases (USPs) [108, 109]; (4) inhibition of tumor angiogenesis: These cytokines suppress tumor vascularization through multiple mechanisms—they downregulate proangiogenic factors (VEGF, bFGF, MMPs), block vascular endothelial cell proliferation, and induce endothelial apoptosis, collectively preventing metastatic spread [110, 111, 112]; (5) suppression of tumor metastasis: disruption of the endogenous type I IFNs signaling pathway using IFN receptor 1 (IFNAR1)^−/−^ mice diminishes antitumor effects of NK cells and accelerates bone metastasis of breast cancer, suggesting that these interferons can inhibit tumor metastasis immune activation mechanisms [113]; and (6) impact on anticancer therapeutics: elevated type I IFNs concentrations are critical for optimizing therapeutic outcomes in conventional therapies, such as chemotherapy and radiation. They can enhance immunogenic cell death (ICD) triggered by these treatment modalities, thereby amplifying their antineoplastic effects [114, 115].

In recent decades, the immunomodulatory functions of type I IFNs in oncological contexts have been unveiled, including their role in eradicating malignant cells, maintaining immunoediting equilibrium with genomically unstable neoplasms, and permitting evasion of tumor clones with attenuated antigenicity [116]. Moreover, these cytokines can enhance the immunogenicity of the TME. They can stimulate antigen‐presenting cells (APCs), augment NK cell cytotoxicity, and enhance T‐cell responses. These immunomodulatory properties constitute a critical determinant of immunotherapy response [117]. Type I IFNs have been shown to significantly modulate the therapeutic efficacy of a myriad of immunogenic therapeutics, including ICD‐inducing agents and ICIs [115, 118]. Type I IFNs, especially IFN‐α and IFN‐β, orchestrate multifaceted functions throughout all phases of cancer immunoediting, establishing them as fundamental regulators of antitumor immune responses [100]. Their broad impacts include direct suppression of tumor proliferation and metastasis, modulation of TME, and enhancement of the overall efficacy of cancer immunotherapy.

## 4 Mechanisms of IFN‐α and IFN‐β in Mediating Immune Responses

IFN‐α and IFN‐β serve as pivotal regulators of innate immune defenses, and they orchestrate responses to infections, malignancies, and other pathological conditions [119]. These cytokines act as a critical intermediary linking innate and adaptive immune responses, offering a multilayered defense mechanism [120, 121]. Their production is tightly regulated, and they are often triggered by specific molecular cues such as nucleic acids, which serve as key danger signals in the pathological context. Once they are produced, IFN‐α/β initiates a signaling cascade that influences diverse cellular processes, critically modulates immune cell activity, contributes to immunosurveillance, and maintains immune homeostasis [122, 123].

### 4.1 Key Stimuli of IFN‐α and IFN‐β Production—Nucleic Acids

**FIGURE 2:**
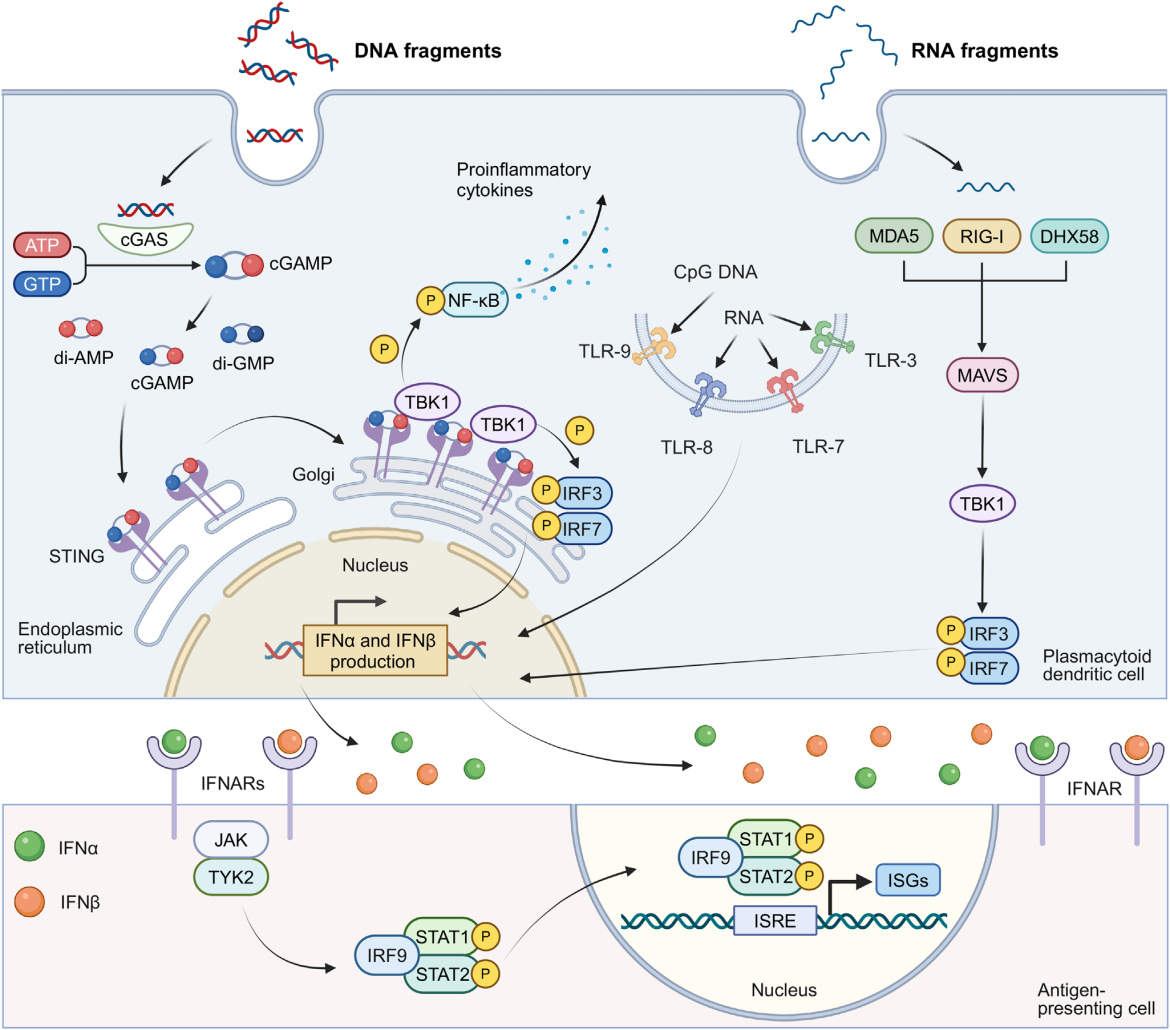
Overview of main pathways for the generation of IFN‐α/β in pDCs and the subsequent signaling pathways in APCs. IFN‐α/β can be produced by recognizing DNA fragments or CpG DNA through the cGAS‐STING pathway or TLR, respectively. Alternatively, their production can be initiated by recognizing RNA fragments via RIG‐I, MDA5, or DHX58, or CpG RNA through TLR3, TLR7, or TLR8. After production, IFN‐α/β bind to membrane‐bound IFNAR receptors, triggering JAK‐STAT signaling cascade activation. This induces the formation of the IFN‐stimulated gene factor 3 (ISGF3) transcriptional complex (STAT1‐STAT2‐IRF9) that translocates to the nucleus to regulate ISG expression. Created with BioRender.com.

IFN‐α and IFN‐β, pivotal members of the type I IFNs family, are secreted by multiple cell types including fibroblasts, such as fibroblasts, leukocytes, and endothelial cells, with pDCs demonstrating particularly robust production capacity [124]. Remarkably, pDCs can synthesize interferons at a more than a hundred‐fold higher level at a significantly faster rate compared with other monocyte‐derived populations [125]. Figure 2 illustrates the primary pathways of IFN‐α/β generation in pDCs and their downstream signaling cascades.

The generation of IFN‐α and IFN‐β is triggered through pattern recognition receptors (PRRs) activation., which is predominantly driven by the recognition of nucleic acids. The vital recognition process is predominantly governed by two key groups of PRRs: (1) cytosolic DNA sensors: upon binding to double‐stranded DNA (dsDNA), the enzyme cGAS synthesizes 2',3'‐cGAMP from nucleotide precursors (ATP/GTP). This endogenous signaling molecule, along with bacterially derived cyclic dinucleotides (CDNs) such as 3′,3′‐cyclic‐di‐GMP (cdG) and 3′,3′‐cyclic‐di‐AMP (cdA), activates STING, an endoplasmic reticulum (ER)‐localized transmembrane adaptor protein. The binding of these CDNs to the STING induces its oligomerization and subsequent relocation to trans‐Golgi vesicles, a process facilitated by the “STING ER exit protein” (STEEP), encoded by CxORF56. After reaching its destination, STING undergoes palmitoylation at specific cysteine residues (Cys88/ Cys91), which activates downstream signaling proteins [126, 127, 128, 129]. This process results in the stimulation of TANK‐binding kinase 1 (TBK1), leading to subsequent phosphorylation of interferon regulatory factors (IRF3/IRF7). TBK1 additionally phosphorylates inhibitor of kB kinase ε (Ikkε), which induces nuclear factor kappa B (NF‐κB) translocation and subsequent expression of inflammatory mediators including IL‐6, TNF‐α, and IL‐1β [130, 131, 132]. TLR9, an endosomal toll‐like receptor (TLR) specific for DNA sensing, also contributes to this pathway by recognizing unmethylated CpG DNA and activating IRF7 [131, 133]. (2) Cytosolic RNA sensors: The cytosolic receptor RIG‐I, containing characteristic caspase recruitment domains and exhibiting helicase/ATPase functionality,

Cytosolic RNA sensors including RIG‐I, which has a caspase‐binding domain and possesses RNA helicase and ATPase activities, can be activated by specific RNA structures to initiate the interferon production pathway [134, 135]. Meanwhile, two RNA helicases, melanoma differentiation‐associated protein 5 (MDA5) and ATP‐dependent RNA helicase 58 (DHX58), can recognize structurally distinct RNAs compared with RIG‐I. Both MDA5 and DHX58, similar to the cGAS‐STING pathway, can initiate TBK1‐IRF3 activation through mitochondrial antiviral‐signaling protein (MAVS)‐mediated signaling [134, 136, 137]. The activation of IRF3 leads to its dimerization and association with protein adaptors, stimulating the IFN beta 1 (IFN‐β1) promoter. Initial IFN‐β1 production triggers stimulation of IRF7, which is instrumental in inducing the transcription of various IFN‐α isoforms [138, 139]. Additionally, endosomal TLRs including TLR3, TLR7, and TLR8 also recognize viral RNA, activating similar signaling cascades that contribute to the generation of IFN‐α/β [140, 141].

Beyond endogenous and pathogen‐derived nucleic acids, small‐molecule STING/IFN agonists—such as 5,6‐dimethylxanthenone‐4‐acetic acid (DMXAA), flavonoid compounds, and CDNs, can trigger IFN‐α/β production, thus displaying effective tumor‐suppressive activity [131, 142, 143]. Furthermore, certain cytotoxic chemotherapeutic agents, including cyclophosphamide and doxorubicin, have been demonstrated to activate type I IFNs pathways [144, 145]. The activation has been observed both in murine models and clinical settings, indicating these chemotherapeutic agents may contribute to the enhancement of interferon‐mediated anticancer responses. The cGAS‐STING signaling axis can be stimulated by diverse nucleic acid species, extending beyond microbial DNA, during type I IFNs biosynthesis. Emerging evidence reveals that the cGAS‐STING axis can facilitate interactions between RNA detection systems (RIG‐I/MDA5/DHX58) and TLR signaling [146, 147]. This cross‐communication mode enables comprehensive immune surveillance against both pathogenic threats and malignant. Therefore, the cGAS‐STING pathway becomes a critical immunological interface linking innate and adaptive responses, particularly in the context of tumor immunotherapy.

### 4.2 Signals Elicited by IFN‐α and IFN‐β

Nucleic acids, along with other molecular agents, induce IFN‐α/β production and elicit a sequence of immunostimulatory actions, including the upregulation of IFN‐stimulated genes (ISGs) transcriptionally [115, 148]. IFN‐α/β mediate their biological functions via dimeric transmembrane receptors, IFNARs, comprising IFNAR1 and IFNAR2 subunits. When IFN‐α/β binds to IFNARs, it activates the JAK‐STAT pathway through tyrosine kinase 2 (TYK2) and Janus kinase 1 (JAK1) kinase activation, subsequently inducing signal transducer and activator of transcription 1 (STAT1)‐STAT2 heterodimer formation [148, 149]. These dimers then combine with interferon‐regulatory factor 9 (IRF9) to form the ISGF3 complex [150]. This complex translocates to the nucleus and binds interferon‐stimulated response elements (ISREs) to activate ISG expression [151]. These ISGs encode a wide spectrum of proteins that mediate diverse effects of type I IFNs. These effects include the inhibition of viral replication, cell cycle regulation, apoptosis induction, and an enhancement in antigen presentation [152].

### 4.3 Effects of IFN‐α and IFN‐β on Immune Cells and Immunosurveillance

In cancer immunotherapy, IFN‐α/β are recognized for their potent immunomodulatory properties, resulting in their beneficial effects on the efficacy of immunotherapy. Primarily, these interferons are crucial in modulating immune responses by acting on diverse immune cells, including (1) DCs: these interferons have the ability to stimulate the activation and maturation of DCs. They enhance the antigen‐presenting capability of DCs, which is crucial to initiate T‐cells activity in response to tumor antigens [153]; (2) T cells: IFN‐α/β are essential for T cells activation and proliferation, such as CD8^+^ cytotoxic T lymphocytes (CTLs) and CD4^+^ helper T cells [154, 155]. In addition, they promote the generation of effector and memory T cell populations that are crucial for sustained antitumor immunity; (3) NK cells: IFN‐α/β enhance NK cell cytotoxic activity, which serves as early defense against tumor cells. They also upregulate activating receptors on NK cells, thereby improving malignant cell recognition and elimination capacity [156]; (4) MDSCs and Tregs: IFN‐α/β can modulate suppressive activities of both MDSCs and Tregs within the TME, potentially counteracting prevalent immunosuppression during immunotherapy [157, 158]; (5) B Cells: the role of IFN‐α/β extends to B cells to enhance their antibody production and antigen‐presentation, which are crucial for effective humoral responses in tumor immunity [159]; and (6) TAMs: IFN‐α/β can promote the polarization of TAMs to steer them toward a phenotype that supports antitumor activities rather than protumor activities including tumor proliferation and metastasis [160].

Beyond their immunomodulatory functions, IFN‐α/β plays a central role in coordinating systemic antitumor immunity [161]. IFN‐α and IFN‐β potentiate immune surveillance mechanisms, enabling more efficient identification and elimination of emerging malignant cells, thereby promoting immune surveillance mechanisms. By upregulating MHC molecules and tumor antigens, IFN–α/β increase tumor cell immunogenicity and promote more efficient CTL activation, thereby facilitating tumor cell elimination [162, 163]. For example, Klement et al. [36] found that myeloid‐derived IFN‐I signaling contributes to patient responsiveness to anti‐PD‐1 therapy (nivolumab). Their research revealed that PD‐1/PD‐L1 interaction between myeloid cells (mPD‐1) and tumor cells (tPD‐L1) activates SHP2, which in turn antagonizes the IFN‐I and STAT1 pathway. This suppression downregulates CXCL9 expression in myeloid cells—a critical chemokine for CTL recruitment—ultimately promoting immune escape at metastatic niches. In addition, type I IFNs exhibit antiangiogenic properties that restrict tumor vascularization and suppress metastatic potential by reducing their exposure to angiogenic factors [164, 165].

The pivotal role of IFN‐α/β in orchestrating anticancer immunity is underscored by their broad effects on various immune cell types through diverse mechanisms. These interferons not only activate and regulate essential immune cells but also modulate the TME to counteract cancer progression. Through coordinated modulation of immune cell activation, expansion, and specialization, type I IFNs ultimately generate potent and multifaceted antitumor immunity, suggesting their potential in immunotherapeutic treatments.

## 5 Clinical Application of Anti‐PD‐1/PD‐L1 Therapy and Resistance Development

Since 2014, clinical use of PD‐1/PD‐L1 blockade has established remarkable therapeutic efficacy. Currently approved agents include PD‐1 inhibitors (nivolumab, camrelizumab) and PD‐L1 blockers (durvalumab, avelumab) for multiple malignancies [166]. However, there is a striking variability in the response to ICIs across between different cancer types and among individual patients with identical diagnoses [167]. High response rates (50%–90%) to ICIs have been observed in Hodgkin's lymphoma, MSI‐H tumors, Merkel cell carcinoma, and desmoplastic melanoma, which are typically characterized by one or more of the following features: elevated PD‐L1 expression, viral etiology, MSI‐H/dMMR, or a substantial mutational burden [76, 168, 169, 170]. In contrast, other cancers, including melanoma, NSCLC, head and neck tumors, gastroesophageal junction tumors, urothelial carcinoma, hepatocellular carcinoma (HCC), and renal cell carcinoma, exhibit a relatively lower response rate to ICIs. A response rate of 35%–40% is seen for melanoma, and a response rate of 15%–25% for other tumors [167, 171, 172, 173, 174].

In addition to the variations in the response rate, the growing challenge of both primary and acquired resistance to ICIs has become a major research focus. Current definitions of anti‐PD therapy resistance remain inconsistent across studies. From a clinical perspective, the Society for Immunotherapy of Cancer (SITC) defines primary resistance as disease progression occurring between 6 weeks (two treatment cycles) and 6 months following ICI initiation. In contrast, acquired resistance refers to disease progression following an initial clinical benefit from the treatment, such as obtaining an objective response or reaching a stable disease (SD), which has been maintained for at least 6 months [175]. In terms of biological mechanisms, primary resistance is developed in tumors showing no initial response to immune checkpoint inhibition, whereas acquired resistance develops in tumors that initially respond to an ICI but later develop refractoriness [10]. This review examines the potential of IFN‐α/β to overcome resistance to PD‐1/PD‐L1 blockade, encompassing fundamental research and translational studies. Table 1 summarizes clinical trials of combined IFN‐α/β and PD‐1/PD‐L1 blockade.

**TABLE 1:** Clinical trials evaluating combined PD‐1/PD‐L1 blockade and IFN‐α/β therapy in cancer treatment.

Type of cancer	Observations	Phase	NCT number	Status	First posted	References
Advanced melanoma, renal cell carcinoma	The combination therapy of pembrolizumab with pegylated IFN‐α2b regimen was more significant and the efficacy appeared to be weak.	Phase Ib	NCT02089685	Completed	March 18, 2014	[176]
Advanced melanoma	Pembrolizumab combined with PEG‐IFN for patients with PD‐1‐naïve metastatic melanoma had acceptable toxicities and displayed favorable clinical outcomes.	Phase Ib/II	NCT02112032	Completed	April 11, 2014	[177]
Advanced or metastatic renal cell carcinoma, metastatic NSCLC, and melanoma	Atezolizumab combined with IFN‐α and other immunomodulatory therapies for advanced/metastatic malignancies.	Phase I	NCT02174172	Completed	June 25, 2014	–
Advanced melanoma	Neoadjuvant treatment with high‐dose IFN‐α‐2b and pembrolizumab showed encouraging clinical efficacy, although it was associated with a high incidence of treatment discontinuation.	Phase I	NCT02339324	Completed	January 15, 2015	[178]
Multiple myeloma, acute myeloid leukemia or lymphoma	To determine the optimal dosage and side effects of recombinant vesicular stomatitis virus (VSV) engineered to carry human NIS and IFN‐β genes, either alone or in combination with cyclophosphamide, ipilimumab, nivolumab, or cemiplimab.	Phase I	NCT03017820	Recruiting	January 11, 2017	[179]
Metastatic melanoma	To assess the preliminary therapeutic effects of IFN‐α/nivolumab combination therapy.	Phase I/II	NCT03638375	Active, not recruiting	August 20, 2018	[180]
HLA‐A2^+^ refractory melanoma	To assess the therapeutic potential of type 1‐polarized dendritic cell therapy integrated with tumor‐selective chemokine modulation (CKM: IFN‐α‐2b rintatolimod and celecoxib) in PD‐1/PD‐L1‐refractory melanoma patients.	Phase II	NCT04093323	Recruiting	September 18, 2019	–
HCC	Investigating combined PD‐1/PD‐L1 blockade and PEG‐IFN‐α2b therapy in advanced HCC adults.	Phase Ib/II	NCT04943679	Recruiting	June 29, 2021	[177]
NSCLC	To evaluate the efficacy of MEM‐288 plus nivolumab in anti‐PD‐1/PD‐L1‐pretreated advanced/metastatic NSCLC patients with primary refractory or first relapse disease.	Phase I	NCT05076760	Recruiting	October 13, 2021	–
Advanced melanoma	To evaluate the clinical outcomes and safety profile of combining recombinant human IFN‐α1b with toripalimab and anlotinib hydrochloride in advanced melanoma cases.	Phase Ib/II	NCT05539118	Not yet recruiting	September 14, 2022	–
Locally advanced or metastatic solid tumors	To determine the efficacy and safety profile of combining MSC‐IFN‐α with immunochemotherapy.	Phase I/II	NCT05699811	Recruiting	January 26, 2023	–

*Note*: Data sources: https://www.clinicaltrials.gov

## 6 Strategies of IFN‐α/β For Improving Resistance to Anti‐PD‐1/PD‐L1 Therapy

**FIGURE 3:**
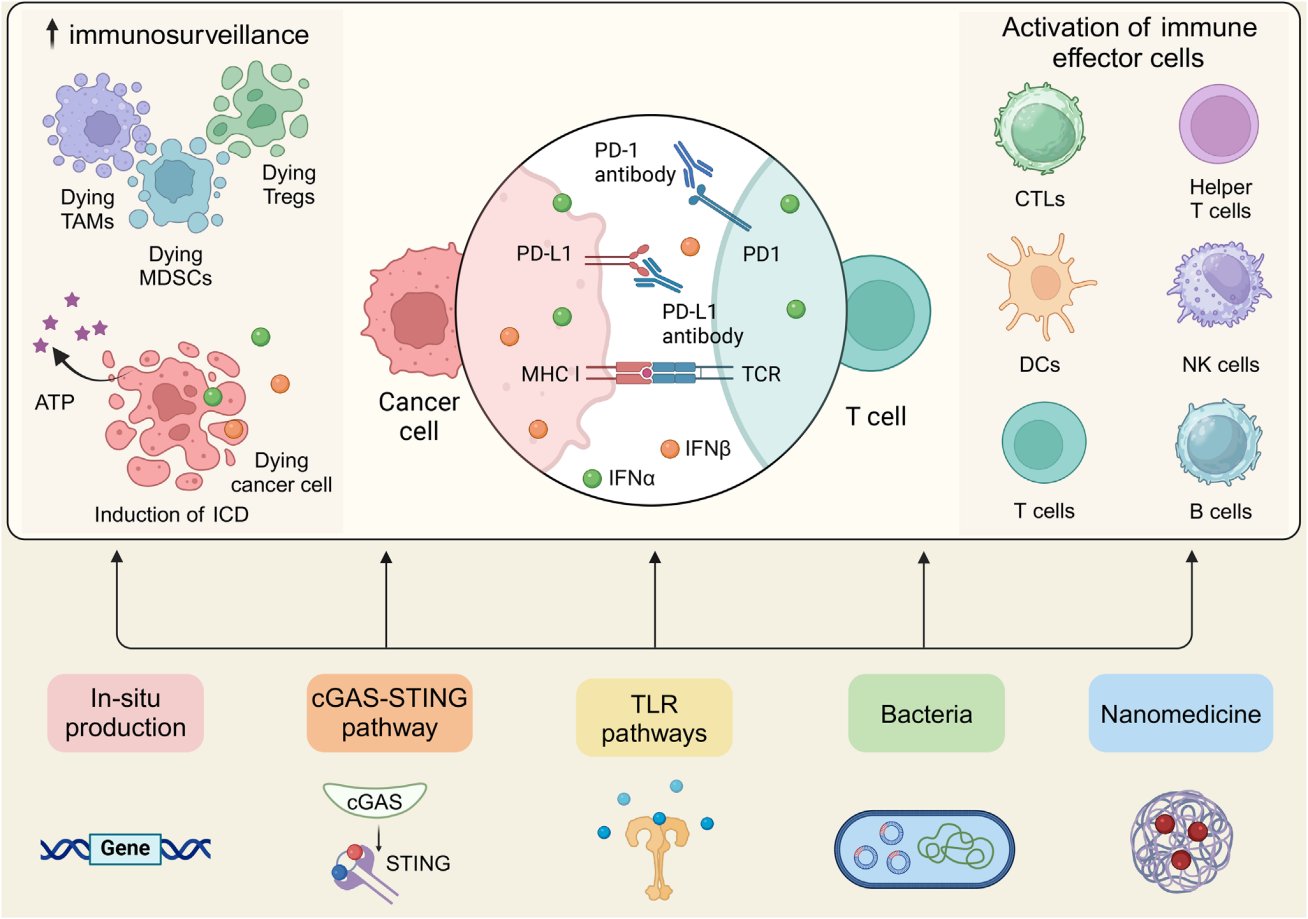
Illustration of therapeutic strategies to apply IFN‐α or IFN‐β for improving resistance of PD‐1/PD‐L1 blockade, demonstrating their dual immunomodulatory effectiveness on immune cell activation and antitumor surveillance enhancement. Created with BioRender.com.

While the molecular mechanisms underlying primary and acquired resistance remain incompletely characterized, numerous strategies have been investigated to overcome immunotherapy resistance. Emerging evidence indicates that IFN‐α/β co‐administration with ICIs can substantially enhance PD‐1/PD‐L1 blockade efficacy. For instance, Hu et al. [181] demonstrated that concurrent administration of IFN‐α and PD‐1 blockade significantly improved outcomes in HCC by altering glucose metabolism. It was revealed that this combination therapy impaired glycolysis in HCC cells while enhancing glycolysis in tumor‐infiltrating CTLs, thereby remodeling the TME to favor an effective antitumor immune response. Meanwhile, IFN‐β has exhibited favorable outcomes combining PD‐1/PD‐L1 blockade. Uehara et al. [182] confirmed that intratumoral IFN‐β at a high dose exerted remarkable antitumor outcomes in a melanoma model. The synergistic combination of IFN‐β and PD‐L1 blockade improved antitumor responses through both immune‐mediated and vascular‐targeted mechanisms. Therefore, combining IFN‐α/β with ICIs presents a promising strategy to overcome PD‐1/PD‐L1 inhibitor resistance, potentially enhancing treatment outcomes for patients. We will delve into the advances in the utilization of IFN‐α/β for overcoming PD‐1/PD‐L1 blockade resistance, with a detailed analysis of their mechanistic roles in improving primary and acquired resistance (Figure 3), and address challenges and opportunities of this approach for enhancing the efficacy of immunotherapy.

### 6.1 Strategies for Improving Primary Resistance to Anti‐PD‐1/PD‐L1 Therapy

Primary resistance, characterized by the initial absence of responsiveness to PD‐1/PD‐L1 inhibition, stems from complex crosstalk between malignant cells, immune components, and the tumor microenvironment [81]. First, differential PD‐L1 expression patterns across tumor and immune cells are important in shaping immunological reactions [183]. Low tumor PD‐L1 levels often confer primary resistance due to the pathway‐specific mechanism of PD‐1/PD‐L1 inhibitors. Second, tumor mutational burden and neoantigen expression on tumor cells are pivotal. Elevated mutation rates correlate with increased neoantigen presentation, enhancing tumor immunogenicity and improving immunotherapy responsiveness [184, 185]. In addition, the constitution and biological status of immunocytes in the TME and peripheral blood significantly influence the immune response. For instance, a high density of CTLs in a TME with immune cell infiltration results in greater responsiveness to anti‐PD‐1/PD‐L1 treatment [185]. Other factors, such as deficient antigen presentation, deactivated critical pathways, intrinsic T cell dysfunctions, epigenetic variations, and tumor heterogeneity, could be accounted for primary resistance [81, 186, 187, 188]. To counteract this resistance, one of the promising approaches involves supplementing PD‐1/PD‐L1 blockade with therapeutic agents to stimulate IFN‐α/β production or unique carriers to enhance the retention of these interferons within the TME.

#### 6.1.1 In Situ Production of IFN‐α/β with Gene Editing

Multiple factors contribute to the diminished or suppressed generation of IFN‐α/β in the tumor microenvironment. Primarily, the breakdown of DNA and antigens in the TME, in conjunction with cGAS‐STING pathway inhibition in malignant cells, hampers innate immune detection and consequent induction for IFN‐α/β production [189, 190]. To address this, strategies for the promotion of intrinsic IFN‐α/β synthesis within the TME using gene editing techniques could be employed to mitigate PD‐1/PD‐L1 blockade resistance [191].

**FIGURE 4:**
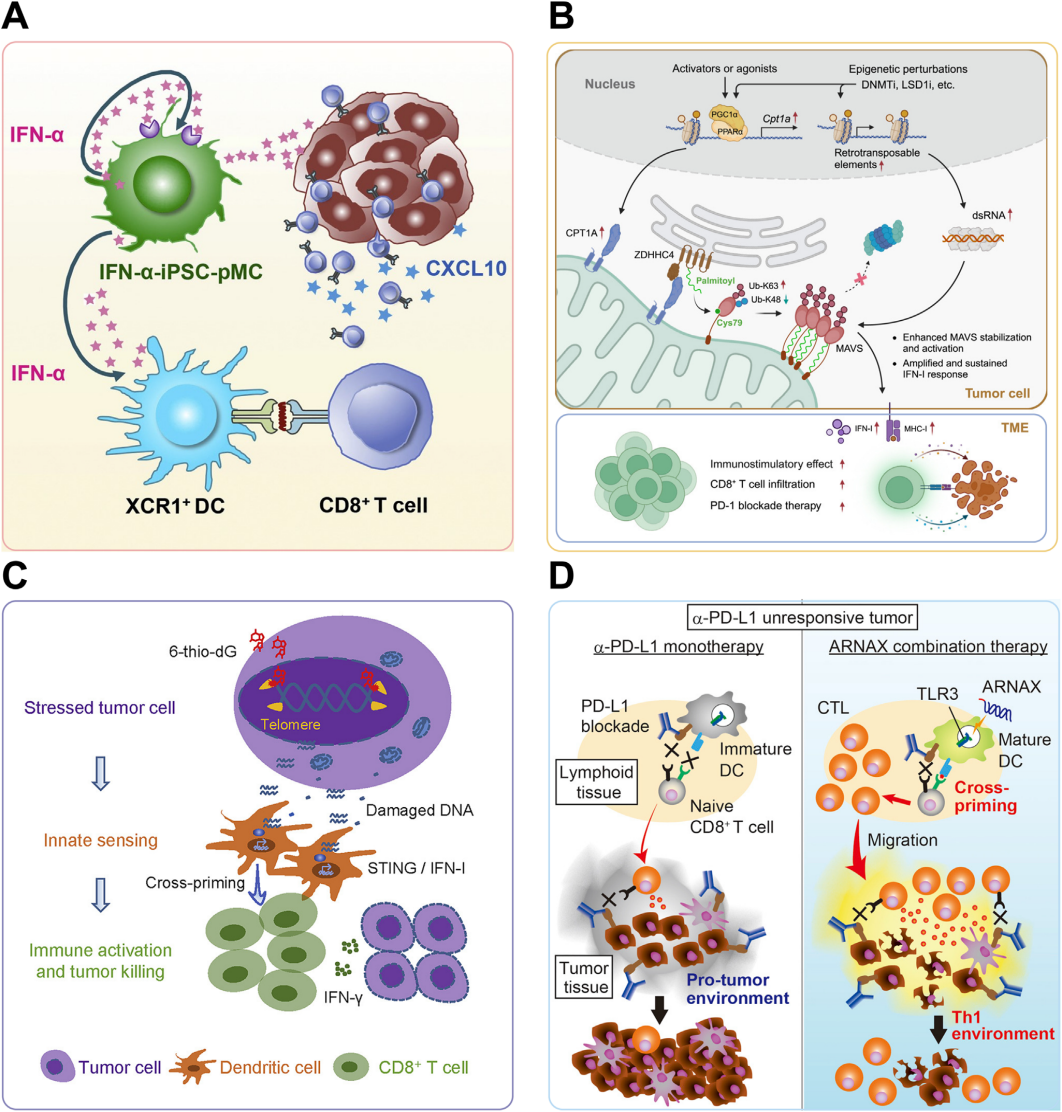
Examples of utilizing gene editing, targeting the cGAS‐STING or TLR pathway to enhance IFN‐α/β production and thereby improve PD‐1/PD‐L1 blockade resistance. (A) Intratumoral administration of IFN‐α‐iPSC‐pMCs stimulates XCR1+DCs, enhancing CD8+ T cell activation and CXCL10 secretion. *Source*: Reproduced with permission from Ref. [192], Copyright 2019, Elsevier Inc. (B) Epigenetic treatment elevates CPT1A, which facilitates the recruitment of ZDHHC4, leading to the palmitoylation and stabilization of MAVS at Cys79. This enhancement promotes a robust type I IFNs response, overcoming resistance to PD‐1 blockade. *Source*: Reproduced with permission from Ref. [197], Copyright 2023, Elsevier Inc. (C) 6‐thio‐dG selectively targets telomerase‐positive tumors by inducing telomeric DNA damage, which stimulates STING signaling, enhances cytotoxic T cell priming and reverses PD‐L1 inhibitor resistance. *Source*: Reproduced with permission from Ref. [205], Copyright 2020, Elsevier Inc. (D) The TLR3‐specific ligand ARNAX, together with TAAs, stimulates the production of antitumor CTLs and establishes a Th1‐type immune response, promoting tumor regression without causing inflammation and successfully overcoming resistance to PD‐1 blockade. *Source*: Reproduced with permission from Ref. [210], Copyright 2017, Elsevier Inc.

In a pioneering study, Tsuchiya et al. [192] innovatively developed IFN‐α‐secreting myeloid cells derived from induced pluripotent stem cells (iPSC) to potentiate immune checkpoint blockade (Figure 4A). These engineered iPSC‐derived proliferating myeloid cells (iPSC‐pMCs) demonstrated stable IFN‐α production. These engineered cells, termed IFN‐α‐iPSC‐pMCs, were shown to effectively migrate to tumor tissues, remodeling the TME and promoting the ISG signature. An array of syngeneic cancer models in mice, including B16‐F10 melanoma, MC38 colon cancer, and CT26 colon cancer, were employed to evaluate the effectiveness of IFN‐α‐iPSC‐pMCs. Remarkably, local administration of these engineered cells both localized tumor regression and systemic CD8+ T cell priming via XCR1+ DC cross‐presentation. Additionally, when combined with PD‐1/PD‐L1 blockade, the IFN‐α‐secreting iPSC‐pMCs demonstrated marked synergistic antitumor activity in preclinical models. This combination therapy effectively overcame the resistance observed in one single treatment and helped establish durable anticancer immunity.

While gene‐edited IFN production shows promise, certain genes are also closely associated with IFN signaling, playing a pronounced role in anti‐PD‐1/PD‐L1 resistance. Research demonstrates that melanocytes expressing the activated *BRAF* oncogene did not exhibit senescence when the IFN signaling was deficient, thereby accelerating the development of aggressive melanomas [193]. The mice with partial resistance to downregulation of IFNAR1 showed delayed melanoma development, reduced metastatic disease, and enhanced responsiveness to PD‐1 inhibitors [193].

The dysfunctional exhaustion state of CD8+ T cells, involving profound loss of proliferative capacity and cytokine production, is disastrous for ongoing viral infections and malignancies. LaFleur et al. [194] found that the phosphatase PTPN2 was a previously unrecognized modulator of this T‐cell differentiation process. Deletion of Ptpn2 enhanced Tim‐3^+^ antitumor responses through the enhanced IFN‐α signaling, leading to the complete elimination of MC38 tumors and enhanced B16 tumor responses to PD‐1 blockade. This study positions PTPN2 as a promising therapeutic target for immunotherapeutic interventions in cancer.

While pembrolizumab is standard first‐line therapy for recurrent/metastatic head and neck squamous cell carcinoma (HNSCC), HPV‐negative HNSCC is notably aggressive and it generally exhibits poor responses to pembrolizumab, with a response rate of only 19%. Nigam et al. [195] found that the SET and MYND domain‐containing protein 3 (SMYD3) was a key regulator of immune evasion in HPV‐negative HNSCC. *Smyd3* depletion simultaneously upregulated both IFN‐α response genes and antigen presentation components. These molecular changes translated to enhanced T cell recruitment and superior response to checkpoint inhibition in vivo, as evidenced by robust CD8+ T cell infiltration and tumor regression.

Elcheva et al. [196] demonstrated that an elevated expression level of IGF2BP1 and IGF2BP3 exhibited significantly lower sensitivity to immune checkpoint inhibitors along with more aggressive disease progression. Specifically, the downregulation of IGF2BP1 in melanoma cells was shown to bolster the growth‐inhibitory influence of IFN signals and enhance the susceptibility of tumors to anti‐PD1 treatment in both murine and human melanoma models. Concurrently, Zhang et al. [197] found that an increase in carnitine palmitoyltransferase 1A (CPT1A) intensified the IFN‐I response, thereby boosting both antiviral and antitumor immunity initiated by epigenetic perturbations and improving responses to PD‐1 inhibitors (Figure 4B). These findings collectively suggest that targeted modifications or pharmacological interventions of specific genes associated with the IFN signaling could enhance type I IFNs production, thus potentially restoring sensitivity to PD‐1/PD‐L1 blockade therapy.

#### 6.1.2 Targeting the cGAS‐STING Pathway

**FIGURE 5:**
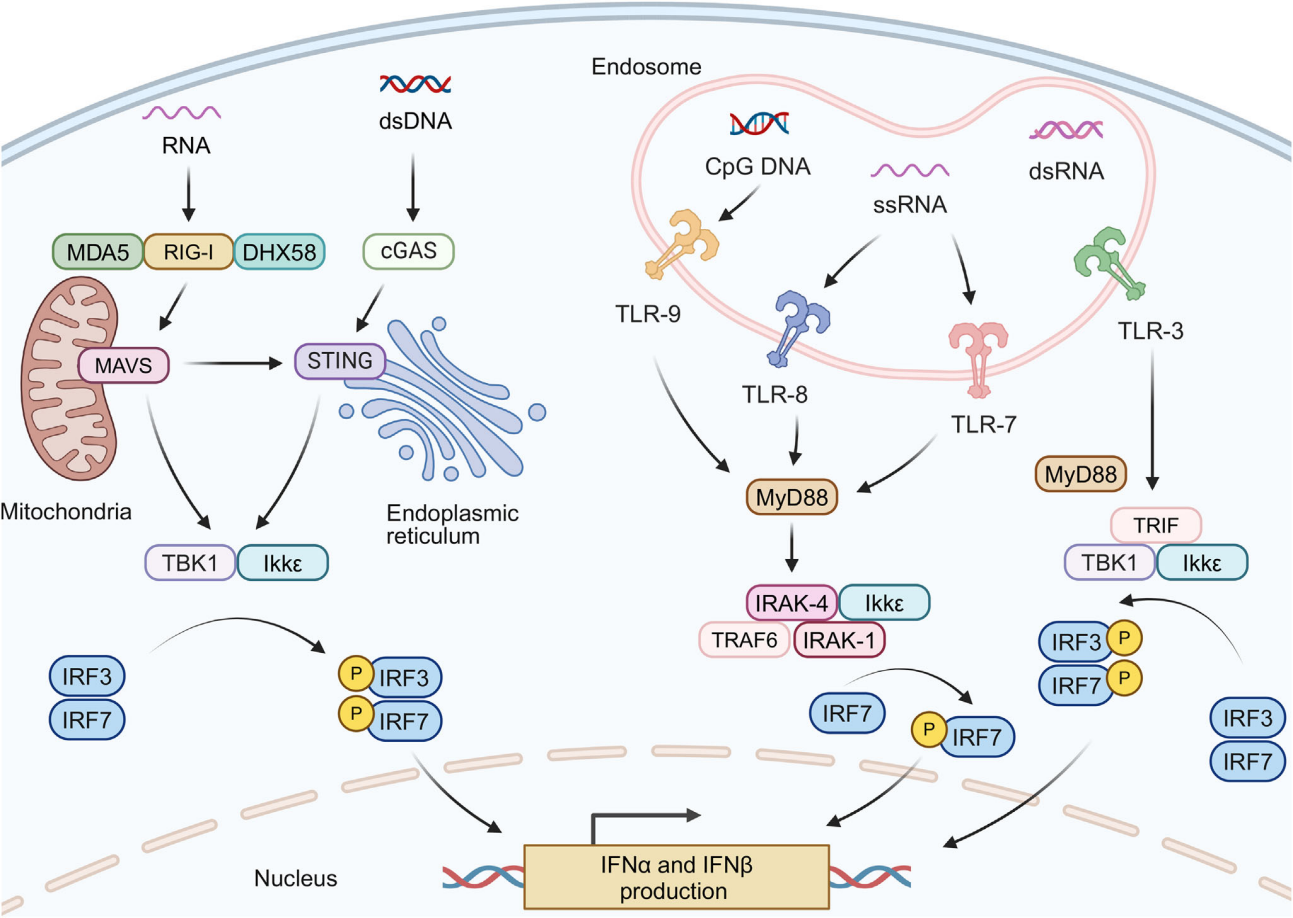
Mechanism of intracellular activation of type I IFNs via cGAS‐STING Pathway and TLRs Pathway. dsDNA is recognized by the cytosolic DNA sensor cGAS, which induces conformational changes and oligomerization of the STING protein on the ER. The activated STING then translocates from the ER to the Golgi apparatus, where it recruits TBK1 and Ikkε, thereby activating the transcription factors IRF3 and IRF7 and inducing the expression of type I IFNs. When RNA is recognized in the cytoplasm by RIG‐I, MDA5, or DHX58, it binds to the MAVS protein on the mitochondria. The activated MAVS then recruits and activates the kinases TBK1 and IKKε, which subsequently phosphorylate the transcription factors IRF3 and IRF7, leading to the expression of type I IFNs. In endosomes, TLR‐3 recognizes dsRNA and activates TBK1 and Ikkε through the TRIF‐dependent signaling pathway, thereby phosphorylating the transcription factors IRF3 and IRF7 to promote the production of type I IFNs. TLR‐7 and TLR‐8 recognize single‐stranded RNA (ssRNA) and, through the MyD88‐dependent signaling pathway, activate IRAK family proteins and TRAF6, leading to the phosphorylation of IRF7 and the promotion of type I IFNs production. Similarly, TLR‐9 recognizes unmethylated CpG DNA and, through the MyD88‐dependent signaling pathway, activates IRAK and IKKε, resulting in the phosphorylation of IRF7 and the induction of type I IFNs expression. Created with BioRender.com.

In addition to utilizing gene editing techniques to induce cells to produce IFN‐α/β in situ, the cGAS/STING pathway and TLRs pathway are the major intracellular pathways responsible for the expression of type I IFNs (Figure 5). Stimulation of IFN‐α/β via the cGAS‐STING pathway represents a key mechanism for augmenting anti‐PD‐1/PD‐L1 therapy efficacy. This pathway can be initiated by multiple stimuli, such as pathogenic infections, abnormal cell replication, DNA damage, and cellular senescence, which result in generating pathological dsDNA [198, 199]. cGAS recognizes this DNA, initiating downstream transcriptional programs for immune activation. The activation is not only crucial for immune defense but also significant in overcoming primary resistance to PD‐1/PD‐L1 blockade.

To activate the cGAS‐STING pathway for strengthening antitumor immunity, small molecular STING agonists as therapeutics candidates have garnered significant attention. Perera et al. [200] investigated the therapeutic potential of MSA‐1, a new STING agonist, in multiple murine tumor models, including immunogenic (LL‐2, B16F10, TC‐1) and nonimmunogenic (4T1) cancers. It displayed low to moderate levels of immunogenicity and a high level of resistance to PD‐1 blockade. Initiation of IFN‐β production by MSA‐1 was instrumental in systemic priming of antigen‐specific CD8^+^ T cells, systemically enhancing antitumor responses. The study demonstrated that intratumoral administration of MSA‐1 resulted in dose‐dependent suppression of tumor progression across the evaluated models. Crucially, IFN‐β induced by MSA‐1 helped converting these immunologically “cold”, nonresponsive tumors into “hot” tumors, thereby sensitizing them to subsequent anti‐PD‐1 antibody therapy. This cold‐to‐hot transformation underlines the potential of STING agonists like MSA‐1 in enhancing the responsiveness of traditionally resistant tumor types to immunotherapeutic treatment. Recent advances in the field have suggested that the STING agonists exert dual functionality‐directly inducing type I IFNs production while simultaneously reshaping the TME. Pancreatic ductal adenocarcinoma (PDAC) represents a prime example of these “immune‐cold” tumors that inherently resist anti‐PD‐1 treatment. Ager et al. [201] demonstrated that localized delivery of the potent STING activator IACS‐8803 into orthotopic models of PDAC successfully orchestrated a shift in the TME. The shift was evidenced by the demonstrated localized delivery of the potent STING activator IACS‐8803. This study revealed that CDNs of varying potency activated or inhibited unique pathways, which could be beyond the traditional paradigm of M2 to M1 macrophage transition. Among these unique pathways was the Myc signaling. Inhibition of the Myc signaling and alterations in metabolic programming indicated a profound and broad‐spectrum influence on tumor‐associated myeloid cells. The revelation of the molecular underpinnings of STING activation supported the multifaceted roles of IACS‐8803 in reversing anti‐PD‐1 resistance. Similar multimodal efficacy is observed with advanced agonists (MK‐1454, E7766) across treatment‐refractory tumors such as bladder and colorectal models, principally through their potent induction of antitumor type I IFNs [202, 203].

Recent investigations indicate that beyond pharmacological STING activation, endogenous factors including specific genetic alterations and telomere instability can modulate cGAS‐STING signaling. Manipulation of these associated factors can stimulate IFN‐α/β production and improve immune tolerance, thus mitigating resistance to PD‐1/PD‐L1 blockade. The enzyme enhancer of zeste homolog 2 (EZH2) is known for its role in transcriptional repression through histone modification, and its overexpression is the culprit for the progression and immune evasion of “cold” prostate cancer (PCa). Morel et al. [204] demonstrated that inhibiting EZH2, either chemically or genetically, triggered a dsRNA‐STING‐ISGs response in PCa cells. This response resulted in the increased expression of genes related to antigen presentation, Th1 chemokines, and IFN‐α/γ production. This immunogenic reprogramming increased tumor‐infiltrating CD8+ T cells and M1 macrophages, overcoming PD‐1 checkpoint inhibitors (CPIs) resistance. Mender et al. [205] investigated the impact of telomere‐targeting drugs on immune response in ICIs resistant cancer cells. A nucleoside analog, 6‐thio‐2'‐deoxyguanosine (6‐thio‐dG), was employed to induce telomere‐associated DNA damage in telomerase‐positive cancer cells. This damage activated dendritic cells through STING/IFN I signaling, enhancing their cross‐presentation capacity and CD8+ T cell stimulation (Figure 4C). The study demonstrated that 6‐thio‐dG potentiated antitumor immunity and reduced resistance to overcome PD‐L1 blockade resistance by simultaneously engaging innate and adaptive immunity in advanced cancers.

#### 6.1.3 Targeting the TLR Pathways

TLRs serve as critical bridges between innate and adaptive immunity. They activate innate immunity and also link it with adaptive immune functions [190, 206]. Specific TLRs (TLR3, TLR7, TLR9) potently induce IFN‐α/β production by activating IRF3/IRF7 transcription factors, thereby amplifying antitumor immune responses through enhanced immune cell recognition and cytotoxicity [207, 208, 209].

To address intrinsic PD‐L1 inhibitor resistance in lymphoma, Takeda et al. [210] employed ARNAX, a TLR3‐targeting adjuvant, in EG7 lymphoma tumors (Figure 4D). ARNAX‐induced DCs maturation via TLR3 signaling is a critical step in generating effective antitumor immunity. This maturation process was pivotal for cross‐priming of antigen‐specific CTLs. The TLR3‐specific adjuvant ARNAX, when co‐administered with tumor‐associated antigens (TAAs), potently enhanced tumor‐specific CTL activation in lymphoid organs and promoted their trafficking to tumors. This combinational approach overcame PD‐1 resistance by engaging the TLR3‐TICAM‐1‐IRF3‐IFN‐β signaling axis in dendritic cells, which proved essential for effective cross‐priming of cytotoxic T lymphocytes.

The clinical landscape has witnessed a surge in the development of TLR7 agonists, which are predominantly administrated in an intratumoral mode. However, a consensus for systemic administration of TLR7 agonists has been reached [211, 212]. This administration route would broaden the scope of treatable cancer types, and cater to therapeutic demands of metastatic cancers. Ota et al. [213] explored the immunomodulatory potential of DSP‐0509, a novel TLR7 agonist, in anti‐PD‐L1‐resistant tumors. The compound activated bone marrow‐derived dendritic cells (BMDCs) to secrete type I IFNs and pro‐inflammatory cytokines, essential for DCs maturation and antitumor T cell priming. In CT26 colon carcinoma models, DSP‐0509 synergized with PD‐1 blockade, achieving superior tumor control while generating effector memory T cells in circulation and tumor tissue.

TLR9 agonists, such as unmethylated CpG oligodeoxynucleotides, are critical for enhancing T‐cell activation and infiltration into tumor sites [214]. A unique attribute of these agonists is their structural mimicry of unmethylated CpG motifs, which are prevalent in bacterial and viral DNA but typically suppressed or methylated in human DNA. These unmethylated CpG oligodeoxynucleotides have been synthesized and administered intratumorally, and they have been demonstrated to effectively trigger innate immune responses [215, 216]. Wang et al. [217] assessed the efficacy of a TLR9 agonist, a CpG oligonucleotide (SD‐101), to overcome anti‐PD‐1 resistance. Intratumoral delivery mediated complete regression of both injected and distant lesions, mediated by robust type I IFN production. The treatment dramatically expanded tumor‐infiltrating CD8+ T cell populations, eventually leading to potent and multifaceted antitumor immune responses.

#### 6.1.4 Bacteria/Bacterial Components in Combination with ICIs

Bacteria and their components that can be recognized by PRRs are harnessed to stimulate type I IFNs responses [218, 219]. Leventhal et al. [220] designed a nonpathogenic *E. coli* Nissle strain to selectively activate STING in tumor‐associated phagocytic APCs while simultaneously engaging complementary innate immune mechanisms. This induction predominantly occurs through TLR‐dependent pathways [221]. This bacterially‐driven IFN‐α/β induction represents an innovative strategy to overcome PD‐1/PD‐L1 blockade resistance.

**FIGURE 6:**
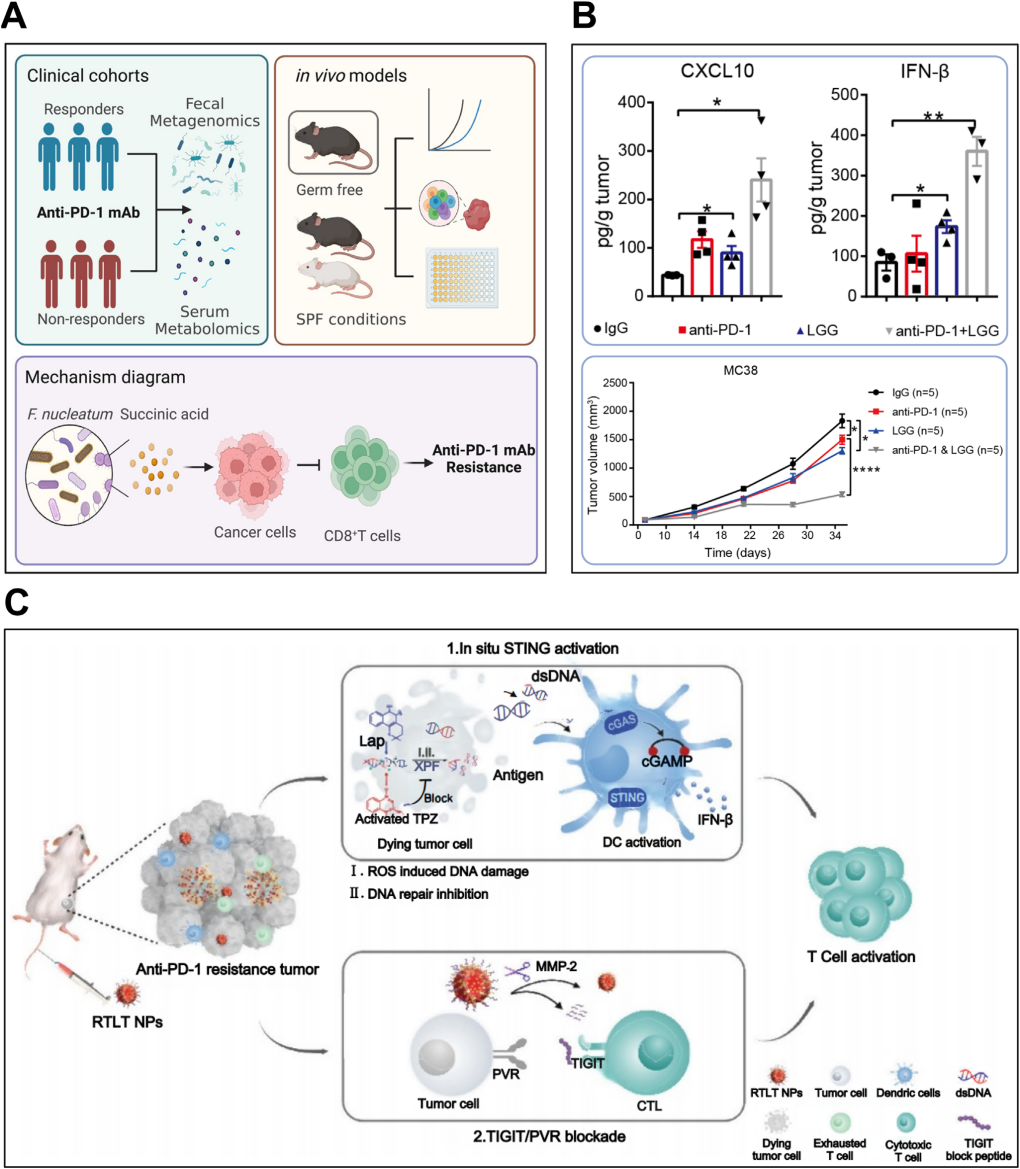
Examples of utilizing bacterial therapies and nanomedicine to enhance IFN‐α/β production and thereby improve PD‐1/PD‐L1 blockade resistance. (A) Elimination of intestinal *F. nucleatum* lowers serum succinic acid concentrations and subsequently reestablishes tumor responsiveness to PD‐1 blockade therapy. *Source*: Reproduced with permission from Ref. [224], Copyright 2023, Elsevier Inc. (B) The LGG+anti‐PD‐1 regimen outperformed PD‐1 monotherapy by elevating IFN‐β and CXCL10 levels (above) while suppressing MC38 tumor progression (below). *Source*: Reproduced with permission from Ref. [225], Copyright 2022, BMJ Publishing Group Ltd. (C) Intravenous delivery of RTLT NPs triggers the STING pathway while simultaneously blocking TIGIT, synergistically improving T cell survival and overcoming PD‐1 blockade resistance. *Source*: Reproduced with permission from Ref. [240], Copyright 2023, Wiley‐VCH GmbH.

PD‐1 inhibitors pembrolizumab and nivolumab are approved for dMMR/MSI‐H metastatic colorectal cancer (mCRC) [222, 223]. However, this target population represents only 5% of mCRC cases, thus the majority of patients exhibit resistance to immunotherapeutic treatment by both PD‐1 inhibitors [223]. A diverse gut microbiome in the unique anatomical position of the colorectal cancer tissue has been found to play a part in its pathogenesis and progression. In this context, Jiang et al. [224] conducted an extensive metagenomic analysis to reveal that a higher prevalence of *Fusobacterium nucleatum* (*F. nucleatum*) in CRC patients was correlated with a lower responsive rate to PD‐1 blockade (Figure 6A). *F. nucleatum* was active in the production of succinic acid, which inhibited the cGAS‐IFN‐β pathway that is pivotal in antitumor immunity. This metabolic disruption led to diminished CD8+ T cell recruitment to tumor sites, accompanied by reduced levels of Th1‐type chemokines CCL5 and CXCL10. Furthermore, the study revealed that succinic acid downregulated the expression of cGAS in CRC cells, thereby blocking responses to IFN‐β and diminishing the effectiveness of immunotherapy. Clinically, eliminating intestinal *F. nucleatum* using metronidazole could re‐store tumor sensitivity to PD‐1 blockade, offering an encouraging strategy to reverse primary resistance in CRC.

Current research suggest that oral commensal bacterial supplementation could modulate antitumor responses of ICIs. Among these bacterial supplements, *Lactobacillus rhamnosus* GG (LGG), a probiotic with widespread use, has been explored by Si et al. [225]. They have uncovered the mechanisms of LGG to ameliorate primary resistance to PD‐1 inhibitors (Figure 6B). Oral delivery of live LGG reshaped the gut microbiome population, specifically enriching immunostimulatory species (*L. murinus* and *B. uniformis*) critical for DCs activation and CD8^+^ T cells’ recruitment. Through a MyD88‐independent mechanism, LGG stimulated cGAS‐STING‐TBK1‐IRF7 signaling in DCs, inducing IFN‐β and CXCL9/10 production that synergistically enhanced PD‐1 blockade efficacy.

#### 6.1.5 Nanomedicine

With the development and integration of different disciplines, the utilization of nanotechnology in the treatment of cancer, in the form of nanomedicines, has received widespread attention [226, 227, 228, 229]. Successful clinical translation of nanomedicines is mainly attributed to their significant advantages in prolonging the in vivo half‐life of drugs and reducing their systemic toxic side effects [230, 231, 232]. Nanomaterials are used as nanocarriers to effectively encapsulate, adsorb, or covalently bind to both hydrophilic and hydrophobic drug molecules because they possess several advantageous properties, including a large specific surface area, a small size, a customizable shape, and ease of modification [233, 234, 235, 236]. A few studies have shown that these nanoparticles or nanomedicine delivery systems for targeting type I IFNs can exploit the enhanced permeability and retention (EPR) effect to accumulate at tumor sites. Once at the tumor, their subsequent induction of IFN‐α/β production enhances the immunogenicity of PD‐1/PD‐L1 inhibitor‐resistant tumors [237, 238, 239].

Direct delivery of IFN‐α/β through nanomedicine to improve immune resistance is an effective strategy. To extend local retention of IFNs and reduce their systemic exposure and associated adverse effects, Lutz et al. [191] introduced an innovative strategy by conjugating IFN‐α and IFN‐β to aluminum hydroxide (alum) particles. To assess long‐term resistance, mice achieving remission were subsequently rechallenged with B16F10 tumors. The mice treated with a combination of αPD1 and IFNs‐anchored alum demonstrated significantly reduced resistance to the rechallenge. The enhanced memory response was ascribed to the dynamics of T cells. Specifically, the treatment with αPD1 and IFNs significantly increased the frequency of memory precursor effector cells (MPECs) within the tumor, which are essential for developing lasting immunity. However, the mice that were treated with the control displayed strong resistance upon rechallenge, and very few survived after a second tumor exposure.

To explore the role of nanomaterials in reshaping the immunosuppressive microenvironment to improve cancer immunotherapy, Zhang et al. [240] developed a novel biomimetic nanoplatform, named RTLT (β‐lapachone and tirapazamine, two cascade‐activating chemotherapeutic agents, were encapsulated within glutathione‐responsive liposomes) (Figure 6C). The liposomes were anchored to a detachable T‐cell immunoglobulin and immunoreceptor tyrosine‐based inhibitory motif domain (TIGIT) block peptide and cloaked with red blood cell membranes. This design allowed the RTLT nanoparticles to efficiently accumulate at tumor sites, evading premature clearance. Upon reaching the TME and exposure to an elevated level of matrix metalloproteinase‐2 (MMP‐2), the TIGIT peptide was released, thereby reversing T‐cell exhaustion and restoring antitumor immunity. Concurrently, the RTLT nanoparticle platform co‐delivered two DNA‐damaging agents that simultaneously impaired repair mechanisms, triggering STING‐dependent IFN‐β production. This approach overcame PD‐1 resistance in breast and colorectal cancers while establishing protective immune memory against metastasis and recurrence.

The identification of the STING signaling cascade has spurred the development of innovative nanomedicine platforms designed to harness this pathway for enhanced antitumor immunity. One popular strategy is the modification of nanocarriers to enhance their targeting function, which helps address many challenges of current tumor immunotherapy [241, 242, 243, 244]. Therefore, nanodrug delivery systems incorporating STING agonists to boost IFN‐I responses have great potential for clinical research and translation. For example, Nakamura et al. [245] evaluated the efficacy of STING agonist‐loaded lipid nanoparticles (STING‐LNPs) in overcoming PD‐1 blockade resistance by activating liver‐resident macrophages for IFN‐β secretion, which propagated NK cell stimulation. IFN‐γ from PD‐1‐expressing NK cells then modulated tumor PD‐L1 expression, creating a self‐sustaining cycle that restored NK cytotoxicity and checkpoint inhibitor efficacy in refractory melanoma. Nanocarriers can be engineered to co‐deliver STING agonists along with other small molecule drugs, amplifying cGAS‐STING pathway stimulation and effectively improving PD‐1/PD‐L1 inhibitor resistance. Liu et al. [246] engineered pH‐responsive TME‐responsive nanoparticles (PMM NPs) to co‐deliver monophospholipid A (MPLA) (TLR4 agonist) and Mn_3_O_4_ (STING agonist) specifically to acidic tumor microenvironments. This combinatorial approach robustly activated NF‐κB and type I IFN pathways (particularly IFN‐β), while reprogramming immunosuppressive elements—reducing Tregs/M2 macrophages and promoting M1 polarization. When paired with anti‐PD‐1, PMM NPs overcame checkpoint resistance in both immunologically “hot” (colorectal) and “cold” (metastatic breast) cancers.

### 6.2 Strategies for Improving Acquired Resistance to Anti‐PD‐1/PD‐L1 Therapy

Acquired resistance to PD‐1/PD‐L1 blockade, or secondary resistance, has recently attracted great interest, while it remains less comprehensively understood compared with primary resistance. This type of resistance often involves tumor evolution, where genetic mutations in critical pathways disrupt the efficacy of checkpoint blockade responses [81, 247]. Whole‐exome sequencing analyses of paired melanoma samples identified JAK1/2 mutations as genomic drivers of tumor progression during pembrolizumab treatment, revealing a key mechanism of acquired resistance [12]. In addition, disruption of the MHC I presentation mechanisms, as evidenced by mutations in β_2_‐microglobulin (B2M), contributes to acquired resistance by cloaking tumor cells from T cell surveillance [12, 248]. Another characteristic of acquired resistance is the loss of neoantigen expression, which could result from genetic alterations or clonal selection within the tumor [249]. This loss facilitates tumor immune evasion by reducing T‐cell recognition. Given the multifactorial nature of acquired resistance to ICIs—where initially responsive tumors develop heterogeneous escape strategies—therapeutic induction of IFN‐α/β signaling emerges as a promising approach to restore PD‐1/PD‐L1 inhibitor sensitivity.

Utilizing CRISPR/Cas9 gene editing, Torrejon et al. [186] developed JAK1/2‐deficient cellular models to recapitulate the molecular mechanisms underlying PD‐1 blockade resistance in both human and mouse systems. This genetic alteration resulted in very low/no responses of these tumor cells to the antitumor effects typically induced by type I IFNs. To counteract this resistance, they employed a combination therapy involving intratumoral injections of a TLR9 agonist and systemic PD‐1 checkpoint blockade. The TLR9 agonist activated pDCs, which trigger their characteristic IFN‐α production, thereby eliciting potent antitumor immune responses to overcome resistance induced by JAK1/2 knockouts. As mentioned earlier, deficient MHC I expression represents a fundamental pathway for tumor immune evasion and acquired resistance to ICIs. To boost immune system antitumor activity, Wolf et al. [250] assessed the combined effects of a STING agonist (CDN) and an interleukin‐2 superkine (IL‐2 superkine, H9‐MSA) on tumors with very low MHC I expression. CDN administration elicited the production of a substantial level of IFN‐β within these MHC I‐deficient murine tumors, activating NK cell‐mediated cytotoxicity. While anti‐PD‐1 monotherapy failed to improve survival, the triple combination of CDN, H9 superkine (sustaining NK cell activity), and PD‐1 blockade demonstrated superior efficacy over dual therapy, suggesting a promising approach to combat acquired resistance.

## 7 Mechanisms of Alleviating Resistance to Anti‐PD‐1/PD‐L1 Therapy by IFN‐α and IFN‐β

Building upon the established roles of IFN‐α and IFN‐β in antitumor immunotherapy and their pivotal contributions to overcoming PD‐1/PD‐L1 blockade resistance, we dive into key mechanisms of enhancing immunotherapeutic efficacy by these interferons. Major mechanisms will be covered, including (1) the cGAS‐STING‐IFN‐I axis, a critical pathway associated with sensing nucleic acids to activate immune response; (2) improvements in tumor immunogenicity; (3) stimulation of immune effector cells; and (4) modulation of the tumor microenvironment. By synthesizing these insights, we provide a comprehensive elaboration of how these mechanisms can be strategically leveraged to overcome resistance as well as improve the clinical efficacy of checkpoint blockade therapies.

### 7.1 cGAS‐STING‐IFN‐I Axis

Cytosolic DNA of cancer cells can be detected by the cGAS, activating robust IFN responses via a stimulator of the STING pathway [251]. It has been found that micronuclei generated through DNA damage or cell cycle defects release genomic material into the cytosol, engaging the cGAS‐STING pathway. Subsequent IFN‐I upregulation enhances immune‐mediated tumor clearance [252]. However, to evade this DNA sensing mechanism, tumor cells often acquire mutations to disrupt the cGAS‐STING axis and induce mutations or deletions in type I IFNs motif [253]. In human colorectal and melanoma cell lines, a few mechanisms have been identified for aberrant cGAS‐STING signaling, including disruption of STING trafficking to the Golgi, epigenetic silencing via promoter hypermethylation, and downregulation of cGAS/STING protein expression. These issues lead to reduced type I IFNs expression and lower levels of downstream ISGs [254]. As previously discussed, STING agonists have shown promise in augmenting the therapeutic effects of PD‐1/PD‐L1 blockade, especially when primary resistance is developed to the therapy.

### 7.2 Other Mechanisms of Alleviating Resistance to Anti‐PD‐1/PD‐L1 Therapy by IFN‐α and IFN‐β

**FIGURE 7:**
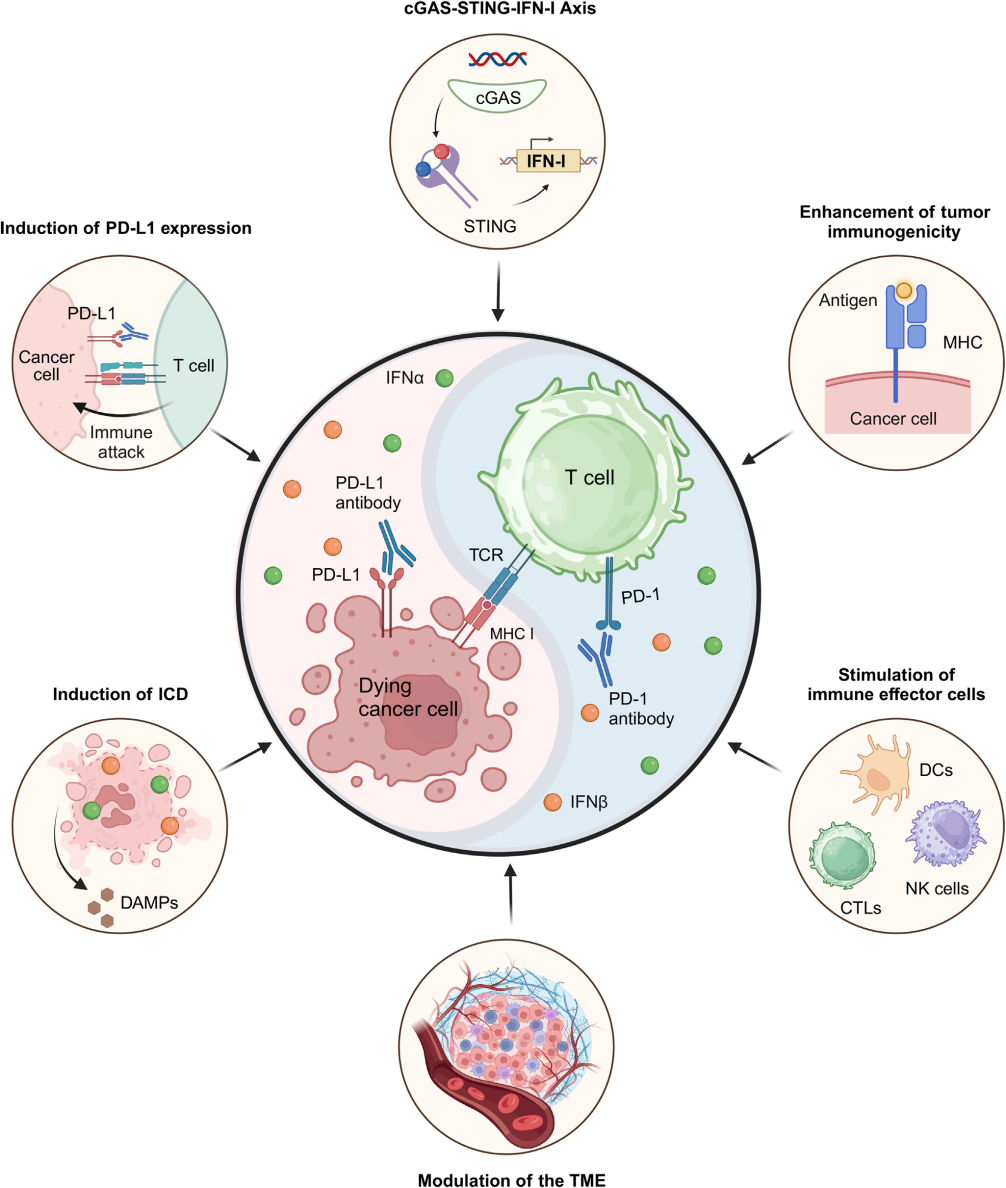
Six mechanisms of alleviating resistance anti‐PD‐1/PD‐L1 resistance by IFN‐α and IFN‐β. In these mechanisms, the “yin‐yang balance” is disrupted, thus inhibiting T‐cell‐mediated tumor cytotoxicity. Restoring this equilibrium enhances T cell cytotoxicity and reinvigorates antitumor immunity, improving checkpoint inhibitor efficacy. Created with BioRender.com.

Studies have shown that administering cGAMP and other agents that boost type I IFN production significantly improves the ability to improve PD‐1/PD‐L1 blockade resistance. In addition to their direct involvement in the cGAS‐STING‐IFN‐I signaling pathway, IFN α/β can mediate a variety of antitumor immune responses to overcome immunotherapy resistance, thereby enhancing the effectiveness of PD‐1/PD‐L1 blockade, as illustrated in Figure 7: (1) Enhancements in tumor immunogenicity: IFN‐α and IFN‐β potently stimulate the surface expression of MHC and tumor antigens on malignant cells, and they become recognizable by the immune system [154, 255]. This heightened visibility can potentially overcome the resistance of anti‐PD‐1/PD‐L1 therapy by enabling T cells to rapidly identify and enable comprehensive tumor clearance. (2) Stimulation of immune effector cells: As detailed in Section 3.3, IFN‐α and IFN‐β enhance antitumor immunity by upregulating chemokines and adhesion molecules, facilitating immune cell infiltration into the TME, including CTLs, DCs, and NK cells, which are crucial for effective antitumor responses [256, 257]. (3) Modulation of the TME: Modulation of the TME can potentiate immunotherapy efficacy. IFN‐α/β have been found to modulate the TME by inhibiting angiogenesis and reducing suppressive activities of Tregs and MDSCs [258, 259], which converts immunologically silent tumors into inflamed microenvironments, creating favorable conditions for checkpoint inhibitor response. (4) Induction of ICD: Exposure to IFN‐α/β stimulates tumor cells to undergo ICD, characterized by the extracellular release of ATP, tumor‐specific antigens, and danger‐associated molecular patterns [108, 260]. These molecules can further stimulate DCs and enhance T‐cell responses, creating an immunogenic TME favorable for anti‐PD‐1/PD‐L1 therapy. (5) Induction of PD‐L1 expression: While IFN‐α/β paradoxically upregulates PD‐L1 expression on tumor cells, this apparent contradiction may actually prime tumors for enhanced anti‐PD‐1/PD‐L1 responsiveness. The IFN‐mediated PD‐L1 induction creates a biological target for checkpoint inhibitors while simultaneously converting immunologically “cold” tumors into “hot,” treatment‐sensitive microenvironments through complex immunomodulatory effects [261, 262, 263].

## 8 Challenges and Perspectives

While anti‐PD‐1/PD‐L1 therapies have transformed oncology, their clinical utility remains limited by pervasive resistance [264, 265]. Our analysis dissects the molecular basis of both primary and secondary resistance and evaluates emerging strategies to circumvent these barriers. More mechanisms for immune therapy responsiveness and resistance will be discovered, and effective PD‐1/PD‐L1 blockade treatments for targeted patients can be optimized by comprehensively considering the factors associated with these mechanisms. Based on the mechanisms identified so far, combination therapy is a primary approach to improving ICIs efficacy [266, 267, 268].

IFN‐α and IFN‐β have proven effective in overcoming anti‐PD‐1/PD‐L1 resistance. Type I FNs serve as master regulators bridging innate and adaptive antitumor immunity through multimodal immunostimulatory effects. However, their role in oncology is complex [269]. Persistent type I IFNs activity upregulates immunosuppressive mediators (IDO, IL‐10) in APCs and T cells, paradoxically driving T cell dysfunction and facilitating immune escape [270, 271]. Prolonged stimulation can also foster feedback inhibition mechanisms, resulting in immune exhaustion and other detrimental effects that may allow cancer cells to evade immune clearance. Professor David G. Brooks and his research team at the University of Toronto analyzed three different cohorts of patients undergoing anti‐PD1 therapy [270]. They found that measuring type I IFN responses in preoperative peripheral blood, specifically through immune protein ISP production, may serve as a prognostic indicator for both initial responses to PD‐1/PD‐L1 blockade and durable clinical outcomes. However, a higher response rate of preoperative type I IFNs for the production of ISP was also found to be correlated with reduced efficacy in PD‐1 antibody treatment and shorter survival periods.

After reviewing the literature, we identified several paradoxical pathways through which type I IFNs can influence pathological processes in cancer development, including (1) disruption of STING or IFNAR signaling attenuates type I IFNs secretion, compromising DC‐mediated T cell activation while promoting regulatory T cell accumulation in the TME [193]; (2) while accumulated mutations or radiation‐induced DNA damages stimulate responses of type I IFNs via cGAS‐STING/ISG activation, cancers often acquire mutations in IFN receptors or downstream effectors to evade this immune surveillance [272]; (3) mutual activation of type I IFNs and p53 induces growth arrest and apoptotic cell death in malignancies, but tumors frequently evade this dual control through p53 mutations [273, 274]; (4) activated STING in senescent cells upregulates the type I IFNs expression, especially within the senescence‐associated secretory phenotype (SASP) compartment. SASP can drive tumor growth when senescent cell replication is paralyzed [275, 276]; (5) type I IFNs regulate the mammalian target of rapamycin complex 1 (mTORC1), influencing glycolysis and autophagy, which can enhance tumor cell growth and survival [277, 278]; (6) type I IFNs can promote epithelial‐mesenchymal transition (EMT) and inflammation at distant sites, thus facilitating metastasis [279].

Therefore, we propose future directions for the development of Type I IFNs: (1) optimized population strategy: With the advances in precision medicine, it is feasible to utilize biomarkers to predict responses and resistance to PD‐1/PD‐L1 blockade. Biomarkers, such as immune regulatory genes, proteomic markers, and the density and distribution of infiltrating immune cells including CD8^+^ T cells, could serve as valuable indicators for immunotherapy [280, 281]. Additionally, responses of a patient to type I IFNs before treatment could serve as a guideline for predicting posttreatment survival outcomes [270]; (2) personalized immunotherapy strategies: a type I IFNs‐incorporating therapy for activating T cells, relieving a locally suppressive microenvironment, and stimulating neoantigen production and presentation on tumor cells could be developed on the basis of individualized immune microenvironment characteristics [282, 283]; (3) integration with nanomedicine: the advances in nanotechnology allow the realization of targeted delivery of STING agonists, TLR agonists and other therapeutic agents by nanomaterials to tumors through the EPR effect or in an active targeting manner to activate the production of IFN‐α/β, reduce systemic toxicity and enhance therapeutic efficacy [283, 284]; (4) bacterial systems: beyond their established role in carcinogenesis, bacteria are gaining traction as innovative drug carriers, leveraging both their delivery capacity and intrinsic immunostimulatory pathogen‐associated molecular patterns (PAMPs) to enhance antitumor immunity and potentiate immunotherapy [285, 286]; (5) exploring immune resistance‐associated genes: recent studies have shown that knocking down or overexpressing certain genes can stimulate the generation of IFN‐α/β, which may restore antitumor activities in patients who display a poor response rate to anti‐PD‐1/PD‐L1 antibodies or are resistant to these immune drugs [166, 287]; and (6) unveiling the mechanisms underlying acquired resistance: the currently identified mechanisms of PD‐1/PD‐L1 resistance likely represent only a fraction of the complex molecular interplay governing therapeutic failure, which have challenged the established research models for studying these mechanisms [288]. Since a significant proportion of patients with acquired resistance have been identified in clinical settings, subsequent treatment methods remain to be developed for these cancer patients [78]. Understanding the roles of IFN‐α/β in both established and novel mechanisms is critical for pioneering next‐generation immunotherapy approaches.

## 9 Conclusion

The last 10 years have witnessed transformative advances in cancer treatment, with immune ICIs targeting PD‐1/PD‐L1 and CTLA‐4 pathways demonstrating unprecedented clinical efficacy across multiple malignancies [166, 289]. However, accumulated clinical evidence has confirmed emerging issues of PD‐1/PD‐L1 blockade, including primary and acquired resistance. Both the tumor and the TME influence the effectiveness of ICI treatment. In the field of ICI therapy, unveiling complex mechanisms of ICI resistance and improving the targeting efficiency of ICI treatment could be achieved through uncoding the intertwined effect between tumor cells and their TME.

IFN‐α and IFN‐β, through their signaling pathways, exert antitumor effects by potently activating cytotoxic immune cells and strengthening systemic immune surveillance mechanisms [99, 290]. Both preclinical studies and clinical trials have supported that the combination of type I IFNs (IFN‐α/β) with PD‐1/PD‐L1 blockade can remarkably enhance therapeutic outcomes. The use of IFN‐α/β for strengthening their antitumor effects primarily stems from IFN‐α/β’s immunostimulatory properties. These cytokines mediate critical interactions between tumor cells and immune cells that help overcome resistance mechanisms to checkpoint inhibition [291]. Notably, strategies to further improve treatment efficacy, such as targeting the cGAS‐STING pathway or the TLR pathways, knocking out or activating specific genes, or employing nanomaterials and bacterial/bacterial components, have been explored for stimulating IFN‐α/β production to mitigate primary resistance.

In addition to summarizing the classic mechanisms underlying PD‐1/PD‐L1 blockade enhancement, a few cutting‐edge mechanisms for overcoming resistance to anti‐PD‐1/PD‐L1 therapy by IFN‐α/β are discussed in this review, including stimulation of the cGAS‐STING‐IFN‐I axis, activation of tumor immunogenicity and immune cells, modulation of the TME, induction of ICD, and upregulation of PD‐L1 expression. However, chronic stimulation of producing type I IFNs may induce APCs and effector T cells to express inhibitory factors, leading to exhaustion of their function and promotion of tumor immune evasion. Therefore, identifying specific targets of PD‐1/PD‐L1 inhibitor‐resistant tumors by IFN‐α and IFN‐β and revealing their underlying mechanisms may help formulate rational personalized treatment plans, develop clinical treatment strategies, and advance therapeutic methods toward personalized and precision therapy.

## Author Contributions

This study was conducted under the expert guidance of Jie Chen, Kui Luo, and Jing Wang, who established the research framework. Peng Gao and Xiao Li authored the preliminary draft and created all visual representations. The manuscript underwent rigorous evaluation and improvement by Zhenyu Duan, Yang Wang, and Yinggang Li. Final approval was obtained from all participating authors.

## Conflicts of Interest

The authors declare no conflicts of interest.

## Ethics Statement

The authors have nothing to report.

## Data Availability

The authors have nothing to report.
